# Comparative genomic analysis of toxin-negative strains of *Clostridium difficile* from humans and animals with symptoms of gastrointestinal disease

**DOI:** 10.1186/s12866-016-0653-3

**Published:** 2016-03-12

**Authors:** Piklu Roy Chowdhury, Matthew DeMaere, Toni Chapman, Paul Worden, Ian G. Charles, Aaron E. Darling, Steven P. Djordjevic

**Affiliations:** The ithree institute, University of Technology Sydney, Sydney, 2007 Australia; NSW Department of Primary Industries, Elizabeth Macarthur Agricultural Institute, PMB 8, Camden, NSW 2570 Australia; Institute of Food Research, Norwich Research Park, Colney, Norwich NR4 7UA UK

**Keywords:** *Clostridium difficile*, Toxin-negative isolates, Comparative genomics, CDI, Zoonosis

## Abstract

**Background:**

*Clostridium difficile* infections (CDI) are a significant health problem to humans and food animals. Clostridial toxins ToxA and ToxB encoded by genes *tcdA* and *tcdB* are located on a pathogenicity locus known as the PaLoc and are the major virulence factors of *C. difficile*. While toxin-negative strains of *C. difficile* are often isolated from faeces of animals and patients suffering from CDI, they are not considered to play a role in disease. Toxin-negative strains of *C. difficile* have been used successfully to treat recurring CDI but their propensity to acquire the PaLoc via lateral gene transfer and express clinically relevant levels of toxins has reinforced the need to characterise them genetically. In addition, further studies that examine the pathogenic potential of toxin-negative strains of *C. difficile* and the frequency by which toxin-negative strains may acquire the PaLoc are needed.

**Results:**

We undertook a comparative genomic analysis of five Australian toxin-negative isolates of *C. difficile* that lack *tcdA*, *tcdB* and both binary toxin genes *cdtA and cdtB* that were recovered from humans and farm animals with symptoms of gastrointestinal disease. Our analyses show that the five *C. difficile* isolates cluster closely with virulent toxigenic strains of *C. difficil*e belonging to the same sequence type (ST) and have virulence gene profiles akin to those in toxigenic strains. Furthermore, phage acquisition appears to have played a key role in the evolution of *C. difficile*.

**Conclusions:**

Our results are consistent with the *C. difficile* global population structure comprising six clades each containing both toxin-positive and toxin-negative strains. Our data also suggests that toxin-negative strains of *C. difficile* encode a repertoire of putative virulence factors that are similar to those found in toxigenic strains of *C. difficile*, raising the possibility that acquisition of PaLoc by toxin-negative strains poses a threat to human health. Studies in appropriate animal models are needed to examine the pathogenic potential of toxin-negative strains of *C. difficile* and to determine the frequency by which toxin-negative strains may acquire the PaLoc.

**Electronic supplementary material:**

The online version of this article (doi:10.1186/s12866-016-0653-3) contains supplementary material, which is available to authorized users.

## Background

*Clostridium difficile* is a Gram-positive pathogen that has emerged to become one of the leading causes of infectious diarrhoea in adult humans, securing its inclusion in the ESCAPE group of pathogens [1–4]. *C. difficile* infections range from being asymptomatic to causing mild or severe diarrhoea and occasionally life-threatening conditions such as pseudomembranous colitis and toxic megacolon [1, 5]. However, community-acquired *C. difficile* infection is being reported with increasing frequency [6] and *C. difficile* is also emerging as a pathogen in animals particularly cattle, pigs and horses [5, 7–10]. Molecular epidemiological studies show that infections in humans and animals can share the same ribotype or multilocus sequence type (ST) [11] suggesting that pathogenic *C. difficile* may traffic between humans and animals, although further studies are needed to confirm these linkages.

*C. difficile* is a genetically diverse and globally dispersed species [11–16] having a clonal structure comprising six major clades (clades 1, 2, 3, 4, 5 and C-I). Clade C-I is the most phylogenetically divergent clade and may represent of a new subspecies of *C. difficile* [17]. Clade C-I typically comprise toxin-negative strains of *C. difficile* [17] but toxigenic variants that reside in Clade C-I have recently been described [18]. Representatives from most clades have been associated with CDI in humans and comprise toxigenic strains with A^+^/B^+^, A^−^/B^+^ toxin types [11, 14, 17, 19–22]. Non-toxigenic strains of *C. difficile* are represented in all six clades [11].

Toxin expression is considered mandatory for the development of *C. difficile* disease [23, 24]. Two large clostridial toxins known as toxins A (308 kDa) and B (260 kDa) encoded by *tcdA* and *tcdB* and the genes implicated in regulating their expression (*tcdC*, *tcdE* and *tcdR*) reside on a 19.6-kb pathogenicity locus known as the PaLoc [25, 26]. The PaLoc is replaced by 115/75 base pair non-coding region in toxin negative strains of *C. difficile* [27]. Approximately 20 % of *C. difficile* strains express a third toxin, known as the binary toxin (CDT) [28]. Genes encoding binary toxin (*cdtA* and *cdtB*) and a regulator gene (*cdtR*) are usually located on a locus (CdtLoc) that is physically separated from the PaLoc. A recent study described six toxin-negative (A-/B-) isolates of *C. difficile* that were positive for CDT from patient with symptoms of CDI [28].

Assays that detect toxin genes or the products of their expression dominate laboratory-based tests used to diagnose infections caused by *C. difficile* [29, 30]. Diagnostic tests that target *tcd* genes underestimate the frequency of detection of toxin-negative strains (including those that express binary toxin) in *C. difficile* disease and as such, their role in disease is poorly understood. Phylogenetic studies show that toxin-negative strains of *C. difficile* cluster tightly with toxin-positive isolates belonging to the same ST [17] suggesting that presence and absence of the PaLoc may be one of the major defining features that differentiate toxin-negative strains from toxin producing strains of *C. difficile*. Notably, oral bacteriotherapy with toxin-negative strains or their spores has been used successfully to treat patients undergoing long-term antibiotic regimes and prevent colonisation by toxigenic strains of *C. difficile* [31–33]. The utility of this therapeutic strategy is supported by previous studies in hamsters which showed that exposure of the gastrointestinal tract to toxin-negative *C. difficile* strains prevented colonisation by toxin-positive strains [34, 35]. Interestingly, challenge studies in hamsters have shown that toxin-negative strains can effectively colonise the gut [36, 37] suggesting that toxin production may be of little consequence in determining the success of colonisation of the gastrointestinal tract. Notably, the toxin-negative strain CD1342 (*tcdA*^*−*^, *tcdB*^*−*^, *cdtA*^*−*^ and *cdtB*^*−*^) was reported to elicit an innate immune response in the caecum resulting in neutrophil infiltration, damage to epithelial mucosa and localised haemorrhagic congestion [36]. These findings suggest that virulence factors are carried by *C. difficile* in addition to the known toxins that can induce host pathology.

Studies of toxin-negative *C. difficile* strains have focused on the characterisation of functional binary toxins and their roles in pathogenesis [28, 33, 38]. The binary toxins *cdtA* and *cdtB* have adenosine diphosphate ribosyltransferase activity but their capacity to induce symptoms of *C. difficile* infection remains unclear [39–42]. Several adhesins, ECM-binding proteins, proteases, motility proteins, hydrolytic enzymes and other surface-associated proteins have been described in *C. difficile* and these factors are likely to contribute significantly to the establishment, progression and severity of *C. difficile* disease [11, 43]. Therefore, further studies are needed to examine the pathogenic potential of toxin-negative strains of *C. difficile* and to determine the frequency at which toxin-negative strains may acquire the PaLoc and express toxins.

Studies that seek to understand the evolutionary history of the PaLoc highlight the complex nature of the multiple clade-specific acquisitions that have occurred after clonal expansion of each clade in populations of *C. difficile* [17]. Those studies report homologous and site-specific recombination events as having played an important role in the loss and gain of the PaLoc [17]. The PaLoc is proposed to be a mobile element that can transfer to toxin-negative strains rendering the recipient with the ability to produce clinically relevant concentrations of ToxA and ToxB [44]. Toxin-negative strains are purported to be ancestral to modern *C. difficile* but lateral genetic events complicate phylogenetic interpretation and alternate hypotheses have been proposed [17]. Genomic studies incorporating a greater diversity of toxin-negative strains of *C. difficile* are needed to shed light on their potential to cause disease.

## Methods

### Isolation and culture of *Clostridium difficile*

All *C. difficile* isolates analysed in this study (P29, 5.3, 19.3, 22.1, H3) were obtained from watery diarrhoea stool samples from their respective hosts (Additional file 1: Table S1). The porcine and equine *C. difficile* isolates analysed in this study were sourced in 2008 from different geographical locations in New South Wales, Australia. The porcine isolate P29 was isolated from a stool sample submitted by the veterinarian attending a piglet with severe but non-fatal diarrhoea. The equine isolate H3 was isolated from a live neonatal foal suffering from non-fatal watery diarrhoea. Stool samples were tested with PCR targeting major ETEC virulence genes [45] and common viruses known to cause diarrhoea in neonatal animals and were plated on blood agar plates to select for enteric pathogens. The stool specimens were initially tested for *Escherichia coli*, *Clostridium perfringens* and *C. difficile* using species-specific PCR primers [46]. Briefly, DNA was extracted from 500 μl of stool sample using a FastDNA spin kit (QBiogene, California, USA) and used as a template for PCR using primers specific for *C. difficile* and *C. perfringens* 16S rDNA [46], *tcdA* and *tcdB* genes (see below) and for *E. coli* [45]. To enrich for *C. difficile* 100 μl of each faecal sample was added to 10 ml cooked meat medium (TM0102 Oxoid Australia) and incubated anaerobically at 37 °C for 24 h using the anoxomat system (MART Microbiology B.B., The Netherlands).

Two hundred μl of culture samples that tested positive for *C. difficile* by PCR were transferred (from cooked meat media enrichment broth) into an Eppendorf tube and centrifuged (10,000 rpm, 5 min). The pellet was resuspended in 1 ml of absolute ethanol (room temperature, 2 h with periodic inversions), harvested by centrifugation (10,000 rpm, 5 min), resuspended in brain heart infusion broth (100 μl) and plated onto *C. difficile* selective agar (CC-BHIA + Taurocholate, PP2362 Oxoid Australia). Plates were incubated under anaerobic conditions at 37 °C for 24 h. Colonies morphologically representing *C. difficile* from each plate were selected and sub-cultured onto CC-BHIA + Taurocholate until pure cultures were achieved.

### DNA extraction

For routine PCR, template DNA was extracted with Chelex (BIO-RAD) from 2 ml brain heart infusion broth cultures grown under anaerobic conditions at 37 °C for 48 h. Briefly, cell pellets were obtained by centrifuging (10,000 rpm for 5 min) 200 μl aliquots of liquid culture, washed 2 × with 500 μl of sterile water and resuspended in 200 μl of 6 % Chelex solution made in Tris-EDTA buffer (pH 7.5). The samples were incubated at 56 °C for 20 min, vortexed for 10 s and incubated at 100 °C for 8 min. After incubation, the sample was immediately transferred to ice. One aliquot was stored at 4 °C for routine PCR tests while the other aliquots were archived at −20 °C.

Sequencing-quality genomic DNA was prepared from 2 ml brain heart infusion broth culture of isolates grown under anaerobic conditions at 37 °C for 48 h. The overnight culture was harvested by centrifugation (10,000 rpm for 10 min), washed in sterile PBS and resuspended in 180 μl of lysis buffer comprising 20 mM Tris-HCl, pH 8.0, 2 mM EDTA, 1.2 % Triton X-100 and lysozyme (20 mg ml^−1^) and incubated for 45 min at 37 °C. DNA was isolated using a DNeasy® Blood and Tissue Kit (Qiagen) by adhering to the manufacturer’s instructions for the extraction of DNA from Gram-positive bacteria.

### PCR conditions

*C. difficile* specific 16S rDNA primers, *C.diff-F:* 5′-TTGAGCGATTTACTTCGGTAAAGA-3′ and *C.diff-R:* 5′-CCATCCTGTACTGGCTCACCT-3′ were used for identification and confirmation of *C. difficile* in enrichment as well as pure cultures. The presence of the *tpi* gene (encoding Triose Phosphate Isomerase), *tcdA* gene (encoding Toxin A) and *tcdB* gene (encoding Toxin B) were tested using previously published primer pairs. Conditions for PCR were as described previously [22] with minor modifications. Briefly, PCR was carried out in 25 μl volumes containing 2 μl of Chelex extracted DNA, 2.5 μl of 10 × PCR buffer, 1.5 mM of MgCl_2_, 1 mM of each dATP, dGTP, dCTP and dDTP (Bioline, Australia), 0.5 μM of each primer and 1 U of BioRad Taq polymerase (Bioline, Australia). PCR cycling conditions consisted of an initial denaturation cycle (2 min, 95 °C) followed by 30 cycles of denaturation (94 °C, 1 min), annealing (55 °C, 1 min) and extension (72 °C, 2 min). The cycling process was completed with a final extension of 72 °C for 5 min.

### Whole genome sequencing, data assembly and phylogenetic analysis

Sequencing was performed at the Next Generation Sequencing facility within the ithree institute at the University of Technology Sydney using a bench top Illumina MiSeq® sequencer and MiSeq V3 chemistry. Genomic DNA stocks shipped to the sequencing facility at concentrations between 1.8 and of 3.7 ng μl^−1^ were used as template for the preparation of sequencing libraries. The genomes were sequenced and assembled *de novo* using published protocols [47]. Raw data and assembled genome sequences were submitted in GenBank under the following Bio-project numbers, 5.3: PRJNA232267, 19.3: PRJNA239262, 22.1: PRJNA239264, P29: PRJNA239265 and H3: PRJNA238844.

PhyloSift was used to conduct a phylogenetic analysis of the five *C. difficile* genomes (P29, 5.3, 19.3, 22.1, H3) with nine closed *C. difficile* genomes including strains M120, CF5, M68, 2007855, BI1, CD196, R20291, ATCC43255 and CD630 available in the NCBI genome database on the 18th of December 2014 [48]. FigTree version 1.4.0 (http://tree.bio.ed.ac.uk/software/figtree/) was used to draw phylogenetic trees. Genome sequences of *C. perfringens* (ATCC13124), *Clostridium botulinum* (ATCC19397) and *Clostridium tetani* (E88) were included as outgroups in the analysis. To improve visual resolution of the evolutionary distances between test and reference strains of *C. difficile* the final figure was generated without the out-groups.

For reference-genome based phylogenetic inference, raw Illumina reads from all taxa were mapped to a single reference (strain CD630) using BWA-MEM (ver0.7.9a) (Li unpublished, github commit: 3efc33160c) and consensus sequences generated using the samtools/bcftools (ver0.1.19-96b5f2294a) tool-chain [49]. The complete set of consensus sequences were combined into a multiple sequence alignment. 1,216,986 alignment columns containing unresolved nucleotides (N) were removed using Mothur (ver1.33.3) [50]. A total of 3,073,266 (72 %) polymorphic and non-polymorphic sites were retained for further analysis. The inclusion of invariant sites has been demonstrated to improve accuracy of whole genome phylogeny [51]. Maximum likelihood phylogenetic inference was employed using RAxML (ver8.0.20) [52] with the following options: raxmlHPC-PTHREADS-SSE3 -T 40 -f a -x 2136841 -p 1486312 -N autoMRE -m GTRCAT. Inference was carried out under a general time reversible (GTR) substitution model with an infinite mixture model for substitutional heterogeneity (CAT), following the suggestion of the RAxML user guide for datasets of this size. The CAT approximation has been previously demonstrated to be an accurate and highly efficient alternative to Gamma-distributed rate heterogeneity on data sets with many taxa (73 – 1663) [53, 54]. Confidence in each clade of the Maximum Likelihood tree was estimated using the rapid bootstrap procedure [55] with automatic extended majority-rule criterion (100 bootstraps) and the resulting tree and bootstrap confidence estimates were visualized with FigTree version 1.4.0 (http://tree.bio.ed.ac.uk/software/figtree/).

### Multi locus sequence typing and comparative genomic analysis

The online version of *C. difficile* PubMLST database (http://pubmlst.org/cdifficile/) was used to sequence type the isolates from the assembled genome sequences. The database was also exploited to locate certain genes of interest.

The online version of the RAST annotation server (http://rast.nmpdr.org/) [56] was used to annotate the genomes. The Classic RAST annotation scheme and FigFAM release 70 were used to predict genes (5.3 = RAST-ID 6666666.71923, 19.3 = RAST-ID 6666666.71924, H3 = RAST-ID 6666666.72094, P29 = RAST-ID 1440056.4 and 22.1 = RAST-ID 6666666.72093). Amino acid sequences corresponding to translated peptide products of all open reading frames predicted by RAST [57] from each of the five genomes were used in the ‘all Vs all’ homology search protocol deposited in the github repository as cRBLH (https://github.com/cerebis/crblh/tree/v0.1). The protocol included clustering of predicted peptide sequences using a modified reciprocal best hit method, where simplicity was favoured for the apparent advantage in identifying orthogroups [58]. The all vs. all homology search was carried out with LAST [59–61] using runtime parameters (−T 1 -f 0 -e 100). The best hits were used to generate a directed graph with genes as vertices and best hits as edges. Unidirectional links between any two nodes were then pruned. Sets of disconnected subgraphs were then analysed for weak intra-cluster linkages, which likely represented overlap between partially homologous protein clusters. Each subgraph was subjected to modularity optimisation [62] and further decomposed until modularity scores of constituent elements fell below a given threshold (0.2). The nodes of the resulting subgraphs were then written out as protein clusters. Singletons defined as nodes without a single edge to any other were deemed unique/isolated genes.

Whole genome comparisons were performed using Mauve version 2.3.1 [63, 64] and iterative BLASTn analysis (http://blast.ncbi.nlm.nih.gov/Blast.cgi). Inter-isolate regions of interest identified from genome-wide comparisons using Mauve, BLASTp and protein clustering analyses were analysed further using iterative BLASTn and BLASTp searches. Figures of comparative genomic analysis, including comparisons of the PaLoc, were compiled using locally downloaded version of EasyFig version 2.1 [65].

The genome of the epidemic *C. difficile* CD630 strain was used in whole genome BLASTp analysis (in RAST) with our test *C. difficile* genomes to identify genes that have been correlated with pathogenicity. All genes deemed as candidate alternative virulence genes or genes for which the products could potentially confer pathogenic traits were individually interrogated using BLASTp and setting amino acid alignment cut off set to 100 % of input query sequence to avoid any data extrapolation.

## Results

### Toxin-negative *C. difficile* from animals and humans with clinical disease

The original stool samples and primary enrichment cultures of the stool samples tested negative for *C. difficile* toxins A and B. PCR assays using DNA from enrichment broths tested negative for enteric (other than *C. difficile*) and viral pathogens associated with neonatal diarrhoea. The porcine faecal sample was negative for the enterotoxigenic *E. coli* genes STa, STb and LT and *C. perfringens* and the disease symptomology did not correlate with viral disease as diagnosed by the attending veterinarian. Similarly, the foal sample was tested for *E. coli*, *Salmonella enterica* and rotavirus and none were detected. While gastrointestinal disease was most likely associated with the presence of toxin-negative *C. difficile* we cannot rule out the possibility that disease was caused by unculturable/unknown pathogens present in the gastrointestinal tract of these animals*.* Toxin-negative human *C. difficile* isolates 5.3, 19.3 and 22.1 were collected in the course of routine diagnostic tests for *C. difficile-*associated diarrhoea in patients presenting typical symptoms of the disease at a gastrointestinal clinic in Sydney, Australia in 2008.

Interrogation of the *C. difficile* PubMLST database confirmed that none of the toxin-negative isolates in our cohort (P29, H3, 5.3, 19.3, 22.1) had homologs of the known *C. difficile* PubMLST toxin genes (*tcdA, tcdB, cdtA and cdtB)* confirming our initial diagnostic PCR data for toxin A and B genes (Additional file 1: Table S1). Isolates 5.3 (ST15), P29 (ST109) and H3 (ST29) were distinct from each other and from ST types of Australian isolates included in a recent phylogenetic study of *C. difficile* (Additional file 1: Table S1) [17].

### Phylogenetic analysis of toxin-negative isolates of *C. difficile*

A study of the evolution of the *C difficile* pathogenicity locus (PaLoc) identified an extremely divergent clade C-I that exclusively comprised toxin-negative isolates predominantly of Australian origin [17]. A maximum-likelihood phylogenetic tree using a reference-based, whole genome alignment protocol (see methods section for protocol details) that incorporates both variant and invariant sites of the *C. difficile* genome sequences was used to verify the ancestry of our toxin-negative isolates. Our approach uses approximately 72 % of the *C. difficile* genome for the analysis, considerably more than what was used in the original study [17]. All 73 genomes and the reference genome CD630 used in the previous study [17] as well as additional closed genomes of *C. difficile* (strains 2007855, ATCC43255, BI1, CF5, M68, M120 and R20291 from the GenBank database) were used in our initial phylogenetic analysis. A preliminary phylogenetic tree (Additional file 2: Figure S12) revealed that our genome-based phylogeny was largely congruent with that described in an earlier study [11] and clearly indicated that none of the five genomes that were the subject of our study clustered within the divergent clade C-I.

**Fig. 1:**
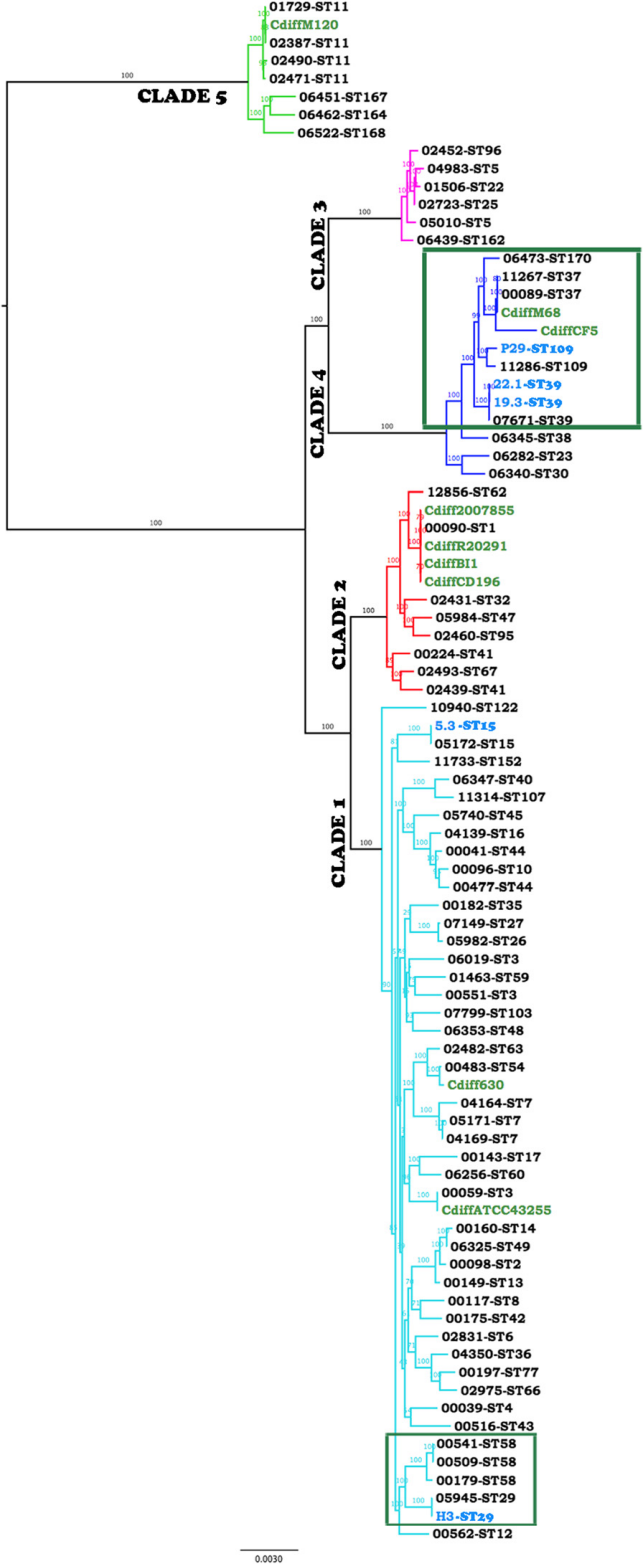
A midpoint rooted phylogenetic tree depicting the five *C. difficile* clades. Australian toxin-negative strains are identified with blue strain names. Complete, closed genomes available in GenBank that were used as a reference are in green text. Genome names in black text are from a previous study [20]. The tree was constructed from reference-based whole genome alignments. Strains within boxed regions indicate genomes used in the ALL vs ALL BLASTp analysis. Clades highlighted in blue indicate clade 4 and in aqua is clade 1

Strains that resided within clade C-I showed greater than 5 % sequence divergence from the reference genome at homologous sites. Clades that have diverged more than 5 % from the reference genome can be poorly resolved in workflows based on Illumina read mapping to the reference [51]. Given this and the uncertain ancestry of the isolates included in clade C-I in the Dingle et al. [11] study, members of clade C-I were excluded from the subsequent analysis based on a cohort of 86 strains depicted in Fig. 1. The branching order of the five clades in our phylogenetic tree was congruent with that reported earlier [17] with identical clustering of strains in the different sub-clades within each of the five clades. The five toxin-negative isolates from our study clustered with strains in Clades 1 (*C. difficile* 5.3, and H3) and 4 (*C. difficile* 19.3, 22.1 and P29) that are known to contain toxin-negative strains [17]. Our toxin-negative isolates (sourced both from animal and human sources) also clustered with strains of the same sequence type that included both toxin-positive and toxin-negative strains isolated from human clinical specimens in an earlier study [17].

Our genome sequences were assembled with a *de novo* assembler using A5 [48]. Prior to conducting a detailed analysis of the toxin-negative *C. difficile* isolates we identified the closest reference genome for tiling genomic scaffolds. A preliminary phylogeny generated using PhyloSift and FastTree (Additional file 2: Figure S2) indicated that *C. difficile* strain CF5 (toxin-positive ST86) was the most appropriate reference to order genomic scaffolds of isolates 19.3 (ST39), 22.1 (ST39) and P29 (ST109) while *C. difficile* strain 630 (toxin-positive, ST54 (PCR ribotype 012) was appropriate to order genomic scaffolds of isolates 5.3 (ST15) and H3 (ST29). Strain CF5 was isolated from a patient in Belgium in 1995 while CD630 is a highly virulent, multiple antibiotic resistant strain of *C. difficile* that caused pseudomembranous colitis in a human patient and later caused an epidemic of *C. difficile* infection in a Swiss hospital ward in 1982. All down-stream analyses of the genomes presented in this study were performed on genomic assemblies with scaffolds ordered to match the reference genomes.

### Homology based functional similarity in the toxin-negative isolates

Initially a Progressive Mauve alignment performed (Additional file 2: Figure S3) on genome sequences of human ST39 isolates 19.3 and 22.1 revealed a high level of nucleotide identity across the genomes with 324 SNP differences. Most of the SNPs were clustered into 7 groups (see Additional file 3: Table S4) suggesting that lateral gene transfer or homologous recombination-mediated genomic rearrangements may be responsible for the differences and were not considered further. Only 27 SNPs were identified that could generate changes in the amino acid sequence of the predicted proteins in the table Additional file 3: Table S4.

To identify genes affected by the SNP changes, a bi-directional BLASTp comparison of 3772 proteins comprising the predicted proteome of strain 19.3 was performed with 3764 predicted protein sequences from strain 22.1 in RAST. The analysis identified 3750 protein sequences that were identical in both the genomes. Nine protein sequences had greater than 99 % sequence identity, six others had greater than 97 % sequence identity and one ORF encoding a hypothetical protein showed 49 % sequence identity (Additional file 3: Table S5). Proteins sharing 97 and 99 % sequence identity predominantly encoded components of the bacterial cell surface including N-acetylmuramoyl-L-alanine amidase, flagellar assembly protein FliH, lipoprotein signal peptidase, putative ABC transporters and permeases. Eight ORFs in isolate 19.3 predominantly encoding hypothetical proteins were missing in isolate 22.1. The genomes of isolates 19.3 and 22.1 comprise 4,181,809 and 4,180,898 bp respectively.

Since isolates 22.1 and 19.3 had high levels of homology, predicted proteins only from isolate 19.3 were included in a pairwise bi-directional BLASTp analysis that seeks to identify conserved genes and major differences among toxin-negative isolates of Australian origin. Isolate P29 had the largest predicted proteome in our collection and was used as the reference. Comparisons of predicted proteomes of human isolates 5.3 & 19.3 and equine isolate H3 with P29 identified major differences in regions harbouring prophage-associated proteins, hypothetical proteins (Additional file 3: Table S5) and putative transposases associated with mobile genetic elements.

### Chromosomal context of the PaLoc insertion site

**Fig. 2:**
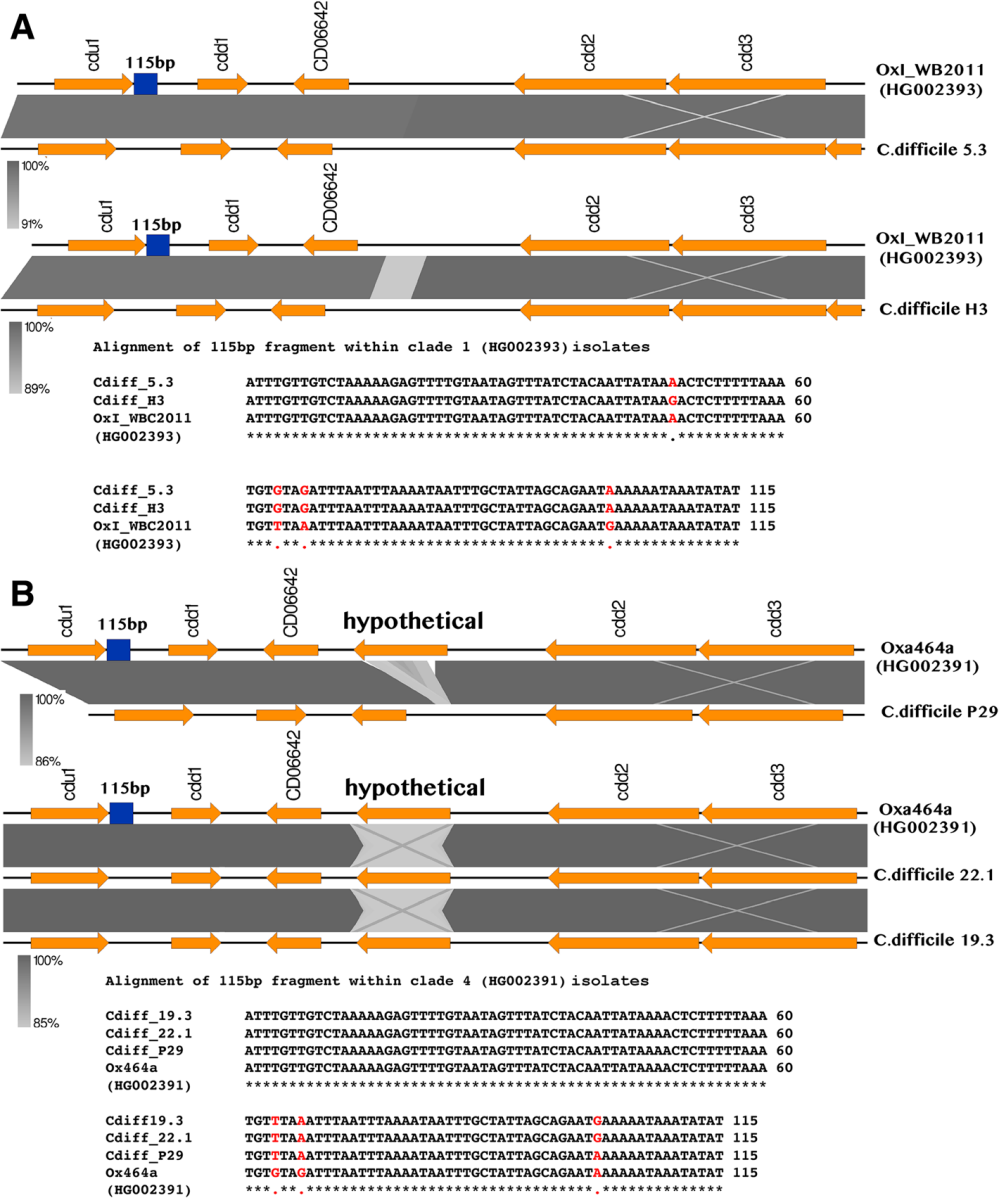
Genetic context of the PaLoc in Australian toxin-negative strains of *C. difficile*. **a** Clade 1 prototype OxI_WB2011 (HG002393). **b** Clade 4 prototype Oxa464a (HG002391)

In toxin-negative strains, a 115 bp sequence replaces the PaLoc locus in phylogenetic clades 1 and 4 and the genetic context in which the 115 bp sequence resides varies within the different clades that represent the *C. difficile* population structure [17]*.* An analysis of the genetic context of the 115 bp sequence in our toxin-negative isolates compared to GenBank sequence entries representing chromosomal regions adjacent to the PaLoc insertion site in toxin-negative strains belonging to Clades 1 (GenBank Accession no HG002393) and 4 (GenBank Accession no HG002391) is depicted in Fig. 2. We identified several SNPs within the 115 bp-conserved fragment and an 80 nt long insertion in strains 5.3 and H3 downstream of the gene designated CD06642 in the reference genomes (Fig. 2). In addition, we identified a 68 nt tandem repeat of the sequence adjacent to the 80 nt insertion site in strain H3 (see Additional file 2: Figure S7). Within members of clade 4, porcine isolate P29 had a deletion of the hypothetical gene seen in the reference region while human isolates 19.3 and 22.1 had a significant decrease in the nucleotide identity in hypothetical genes in the reference genome. Consistent with observations reported earlier, these differences indicate ongoing micro-evolutionary events within the locus that flanks the PaLoc insertion site [17].

### Comparative BLASTp analyses of toxin-negative strains to identify functional similarities between groups of isolates

An all versus all BLASTp based protein-clustering analysis was used to identify the putative core proteome of a subset of Clade 1 strains comprising five genomes and Clade 4 strains comprising 10 genomes (see boxed region in Fig. 1). To maintain uniformity in the input data used in the analysis, raw reads representing each of the genomes from an earlier study [17] were reassembled and annotated using the protocols that were used to assemble and annotate the five toxin-negative Australian isolates of *C. difficile* reported in this study, as described above. The assembly statistics and a comparison of the assembly outputs are presented in Additional file 3: Table S8. On an average, RAST predicted 3700 proteins per genome and these were included in the protein clustering analysis. A bit score cut off of 50 was used to cluster homologous protein sequences. An overview of the data is presented in Table 1.

**Table 1:** Summary of protein clustering results within the different sub-clades containing the five toxin negative isolates included in this study. Summary of protein clusters within the different sub-clades of C. difficile

Clade 1	
Genome names	No of predicted proteins
Core genome	3323
Total number of unique peptides in:	
*C. difficile* C0000509	45
*C. difficile* C0000541	38
*C. difficile* C0000562	176
*C. difficile* C00005945	212
*C. difficile* H3	111
Clade 4:	
Core genome	3357
Total number of unique peptides in:	
*C. difficile* 19.3	8
*C. difficile* 22.1	7
*C. difficile* C00000089	33
*C. difficile* C000011286	86
*C. difficile* C00006473	84
*C. difficile* C00007671	35
*C. difficile* C00011267	60
*C. difficile* CF5	30
*C. difficile* M68	19
*C. difficile* P29	299

Ten clade 4 genomes boxed in Fig. 1 shared 3357 proteins (Table 1). Isolate P29 had the highest number (299) of unshared/unique proteins within the clade 4 cohort and most of the 299 proteins were phage-related (Additional file 3: Table S9). Some of the unique proteins clustered together in the same scaffold indicating lateral movement of phage-associated genomic DNA. *C. difficile* isolates 19.3 and 22.1 carried eight and seven unique proteins respectively. The handful of unshared proteins in 19.3 and 22.1 were attributed to mobile genetic elements or were designated to encode proteins of unknown function. The five *C. difficile* strains within clade 1 shared 3323 proteins. Equine isolate H3 had 111 unique proteins, most of which were phage related or hypothetical with some clustered in single scaffolds (Additional file 3: Table S9).

We queried the viral and prophage database in GenBank with the genome sequences of all five toxin-negative isolates using PHAST [66]. The database confirmed the presence of several regions contain phage DNA in each of the five genomes in Clade 1 (see Additional file 2: Figure S10 and Additional file 4: Table S11). Table 2 lists a summary of the complete prophage sequences (PHAST scores > 110 and over) identified in the five Australian toxin-negative isolates. Notably, isolates 19.3 and 22.1 returned identical phage profiles (Additional file 2: Figure S10). Both genomes carried an identical and complete 56.8 kb phage that is a close relative of phiC2, a 56.5 kb phage first identified in *C. difficile* strain CD242 [67]. phiC2 is one of the first completely sequenced temperate phages in *C. difficile* and regions of phiC2 are present in the majority of *C. difficile* genomes of clinical origin [68]. We also identified a phage in isolates P29 and H3 that carries sequence identity with phiC2. The prophage in P29 was larger (97.4 kb) than the original phiC2 sequence (Additional file 2: Figure S10). Isolate P29 also carries two other complete prophage sequences. One of these, prophage region 9 has significant sequence identity to the *C. difficile* temperate bacteriophage phiCD6356 that belongs to the Siphoviridae family [69]. The Siphoviridae family prophage identified in P29 is 52.8 kb (Additional file 4: Table S11) and is considerably larger than the first report of this phage sequence at 37.6 kb [69]. Isolate H3 also carries an identical variant of phage phiCD6356 (Additional file 2: Figure S12) comprising 41.1 kb. Evidence of other complete prophage genomes is listed in Table 2. Comparative BLASTp analysis of the four genomes (19.3, 5.3, H3 and P29) also aligned to the phage search protocol and confirmed the data generated by PHAST analyses, reinforcing the observation that the major differences in our Australian toxin-negative *C. difficile* isolates have a prophage origin.

**Table 2:** Summary of Phage related regions identified by PHAST in the 5 genomes. Phage sequences identified in this study

PHAST region identifier	Length of prophage	PHAST score	No of predicted CDS	Relative position on genome	Predicted Phage	GC content	Location on Genomic scaffolds
*C difficile* P29 genome							
Region_4	97.4Kb	150	126	1732045-1829500	PHAGE_Clostr_CDMH1_NC_024144	28.7	27.1, 30.1, 40.1, 34.1, 36,1, 5.1, 47.1 and 16.1
Region_8	113.6Kb	150	101	3773336-3886941	PHAGE_Geobac_virus_E2_NC_009552	40.8	5.1, 19.1 and 22.1
Region_9	52.8Kb	140	65	4233532-4286418	PHAGE_Clostr_phiCD6356_NC_015262	29.9	8.1, 26.1, 41.1 and 42.1
Region_10	21.3Kb	100	22	4290994-4312349	PHAGE_Clostr_phiC2_NC_009231	36.4	In over 20 very small scaffolds
*C. difficile* H3 genome							
Region_2	41.1Kb	150	63	925289-966449	PHAGE_Clostr_phiCD6356_NC_015262	28.5	22.1 and 31.1
Region_3	50.1Kb	110	66	1114600-1164714	PHAGE_Clostr_phiC2_NC_009231	28.1	18.1 and 5.1
Region_6	31.5Kb	110	47	4070500-4102080	PHAGE_Clostr_phiC2_NC_009231	29.4	26.1, 27.1, 28.1, 32.1, 33.1
*C. difficile* 5.3 genome							
Region_3	57.9Kb	140	87	1555612-1613526	PHAGE_Clostr_phiC2_NC_009231	28.6	1.1
Region_4	45.1Kb	150	49	1741883-1787019	PHAGE_Clostr_phiSM101_NC_008265	27.2	1.1
*C.difficle* 19.3 genome							
Region_3	56.8Kb	140	74	1700180-1757059	PHAGE_Clostr_phiC2_NC_009231	28.8	1.1
*C. difficile* 22.1 genome:							
Region_3	56.8Kb	140	74	1703858-1760737	PHAGE_Clostr_phiC2_NC_009231	28.8	3.1

Comparative BLASTp analysis of isolates 19.3, 5.3, H3 and P29 identified a 119.3 kb region on contig 11 in P29 (Additional file 5: Table S6). An all versus all protein clustering analysis also identified a subset of unique proteins on contig 11 of the P29 genome but not in the 10 human *C. difficile* genomes within clade 4 (Fig. 1). Equine isolate H3 was not included in this analysis as it was on a different clade. BLASTn analysis of the 119.3 kb region against the *C. difficile* genome database in GenBank identified similarity at the DNA level to parts of *C. difficile* strain CD630 indicating a phage-mediated lateral movement of parts of the genome of CD630 into the genomes of isolates H3 and P29.

### Homology based functional prediction of Putative *C. difficile* virulence factors implicated in host colonization

A homology-based functional prediction analysis of proteins that have been experimentally verified, or predicted to play a role in the colonization of *C. difficile* strain CD630 with homologous proteins in the genomes of the toxin-negative isolates in our study is shown in Table 3 [70, 71]. Most of the proteins i had near perfect (98–100 %) protein sequence identity including proteins encoded by *spo0A*, which serves as a positive regulator for genes required in spore germination and *groEL*, a chaperone that also functions as an adhesin [72]. Several serine-proteases and other metalloproteases which may contribute to the disease development process [70] were also highly conserved. However, some membrane associated proteins that have been shown to play significant roles in the disease development process had lower alignment scores including SlpA, a surface layer protein that is proposed to facilitate host cell adherence [73] and FliC, an adhesin necessary for the colonization of gut epithelium [74].

**Table 3:** Proteins derived from *C. difficile* CD630 that are predicted to play a role in pathogenesis

Selected gene and product	*C. difficile* 630 locus tag	Experimental Verification	RAST Annotation identifiers			
			In *C. difficile* 5.3 (% identity)	In *C. difficile* 19.3 (% identity)	In *C. difficile* H3 (% identity)	*In Cc difficile* P29 (% identity)
Flagellin C gene *fliC*	CD630_02390	yes, RNAseq	fig|6666666.71923.peg.3142 (86)	fig|6666666.71924.peg.3067 (71)*	fig|6666666.72094.peg.3176 (87)	fig|1440056.4.peg.3191 (97)
Flagellin D gene *fliD*	CD630_02370	no	fig|6666666.71923.peg.3140 (88)	fig|6666666.71924.peg.3065 (61)	fig|6666666.72094.peg.3174 (88)	fig|1440056.4.peg.3193 (98)
Precursor S-layer protein gene *slpA*	CD630_27930	yes, proteome	fig|6666666.71923.peg.2081 (43)*	fig|6666666.71924.peg.3725 (59) *	fig|6666666.72094.peg.2466 (58)*	fig|1440056.4.peg.2665 (54)*
Stage 0 Sporulation gene *spoA*	CD630_12140	yes, proteome	fig|6666666.71923.peg.158 (100)	fig|6666666.71924.peg.1561 (99)	fig|6666666.72094.peg.2932 (99)	fig|1440056.4.peg.240 (99)
Fibrinectin binding proten encoding *fbpA* gene	CD630_25920	no	fig|6666666.71923.peg.2930 (99)	fig|6666666.71924.peg.2028 (98)	fig|6666666.72094.peg.3740 (99)	fig|1440056.4.peg.41 (98)
GroEL encoding gene *groL*	CD630_01940	yes, proteome	fig|6666666.71923.peg.3095 (100)	fig|6666666.71924.peg.2995 (99)	fig|6666666.72094.peg.3129 (100)	fig|1440056.4.peg.3232 (99)
Cell surface protein *cwp66*	CD630_27890	no	fig|6666666.71923.peg.2085 (60)	fig|6666666.71924.peg.3721 (78)	fig|6666666.72094.peg.2462 (77)	fig|1440056.4.peg.2669 (79)
Protease *cwp84*	CD630_27870	no	fig|6666666.71923.peg.2087 (98)	fig|6666666.71924.peg.3719 (99)	fig|6666666.72094.peg.2460 (99)	fig|1440056.4.peg.2671 (99)
Adhesin (LPXTG)	CD630_28310	no	fig|6666666.71923.peg.2041 (99)	fig|6666666.71924.peg.3767 (94)	fig|6666666.72094.peg.2504 (98)	fig|1440056.4.peg.2625 (94)
Cell wall binding protein encoding *cwp2*	CD630_27910	yes, proteome	fig|6666666.71923.peg.2083 (98)	fig|6666666.71924.peg.3723 (99)	fig|6666666.72094.peg.2464 (99)	fig|1440056.4.peg.2667 (99)
Cell wall binding protein encoding *cwp12*	CD630_27940	no	fig|6666666.71923.peg.2080 (65)*	fig|6666666.71924.peg.3726 (98)	fig|6666666.72094.peg.2467 (95)	fig|1440056.4.peg.2664 (94)
Cell wall binding protein encoding *cwp11*	CD630_27950	yes, proteome	fig|6666666.71923.peg.2079 (98)	fig|6666666.71924.peg.3727 (99)	fig|6666666.72094.peg.2468 (99)	fig|1440056.4.peg.2663 (99)
Cell wall binding protein encoding *cwp9*	CD630_27980	no	fig|6666666.71923.peg.2076 (99)	fig|6666666.71924.peg.3730 (99)	fig|6666666.72094.peg.2471 (99)	fig|1440056.4.peg.2660 (99)
Cell wall hydrolase (LPXTG)	CD630_01830	no	fig|6666666.71923.peg.3084 (97)*	fig|6666666.71924.peg.2984 (99)	fig|6666666.72094.peg.3118 (100)	fig|1440056.4.peg.3243 (99)
Cell wall binding protein encoding cwp25 gene	CD630_08440	no	fig|6666666.71923.peg.2189 (100)	fig|6666666.71924.peg.3292 (97)	fig|6666666.72094.peg.2128 (99)	fig|1440056.4.peg.522 (97)
N-acetylmuramoyl-L-analini amidase encoding cwp16	CD630_10350	no	fig|6666666.71923.peg.1 (99)	fig|6666666.71924.peg.3716 (65)*	fig|6666666.72094.peg.1495 (99)	fig|1440056.4.peg.1845 (98)
Cell wall hydrolase encoding gene (invasin)	CD630_27680	no	fig|6666666.71923.peg.2107 (99)	fig|6666666.71924.peg.3700 (99)	fig|6666666.72094.peg.2441 (99)	fig|1440056.4.peg.2690 (99)
Polysaccharide de-acetylase	CD630_15220	yes, RNAseq and proteome	fig|6666666.71923.peg.489 (100)	fig|6666666.71924.peg.291 (99)	fig|6666666.72094.peg.3592 (100)	fig|1440056.4.peg.1197 (99)
LmbE-like deacetylase encoding gene	CD630_27900	no	fig|6666666.71923.peg.2084 (93)	fig|6666666.71924.peg.3722 (100)	fig|6666666.72094.peg.2463 (100)	fig|1440056.4.peg.2668 (97)
Invasin/Sh3 domain containing surface protein	CD630_11350	no	fig|6666666.71923.peg.77 (100)	fig|6666666.71924.peg.1641 (98)	fig|6666666.72094.peg.2849 (99)	fig|1440056.4.peg.320 (98)
Cell wall hydrolase/Invasin associated protein	CD630_24020		fig|6666666.71923.peg.2730 (100)	fig|6666666.71924.peg.2779 (98)	fig|6666666.72094.peg.2094 (99)	fig|1440056.4.peg.3691 (99)
Autolysin *acd* gene homolog/mannosyl-glycoprotein endo neta N acetylglucosamine	CD630_13040	no	fig|6666666.71923.peg.256 (100)	fig|6666666.71924.peg.1460 (98)	fig|6666666.72094.peg.3031 (99)	fig|1440056.4.peg.158 (98)
Protease/Serine protease, HrtA family	CD630_32840	no	fig|6666666.71923.peg.1608 (100)	fig|6666666.71924.peg.3459 (99)	fig|6666666.72094.peg.64 (100)	fig|1440056.4.peg.2801 (99)
Intracellular serine protease	CD630_32540	no	fig|6666666.71923.peg.1638 (100)	fig|6666666.71924.peg.3426 (99)	fig|6666666.72094.peg.94 (99)	fig|1440056.4.peg.2834 (99)
Protease/Subtilase family	CD630_07030	no	fig|6666666.71923.peg.2324 (100)	fig|6666666.71924.peg.3155 (97)	fig|6666666.72094.peg.2691 (100)	fig|1440056.4.peg.1169 (97)
Ser-type protease/subtilisin-like serine germination related protease	CD630_22470	yes, Mass spectrometry	fig|6666666.71923.peg.1327 (99)	fig|6666666.71924.peg.2513 (99)	fig|6666666.72094.peg.1937 (99)	fig|1440056.4.peg.1500 (98)
Serine protease precursor/Subtilinase subfamily	CD630_20000	no	fig|6666666.71923.peg.1085 (100)	fig|6666666.71924.peg.2267	fig|6666666.72094.peg.1687 (99)	fig|1440056.4.peg.2329 (99)
Membrane-associated zinc metalloprotease/M50 family peptidase	CD630_21290	no	fig|6666666.71923.peg.1209 (100)	fig|6666666.71924.peg.2404 (99)	fig|6666666.72094.peg.1813 (100)	fig|1440056.4.peg.1610 (100)
Zinc Protease/M16 family peptidase	CD630_26610	yes, proteome	fig|6666666.71923.peg.2996 (100)	fig|6666666.71924.peg.626 (99)	fig|6666666.72094.peg.3677 (100)	fig|1440056.4.peg.438 (99)

* indicates gaps in alignment of amino acid sequences with reference, likely suggesting presence of inactive proteins or variants in the test genomes

## Discussion

*C. difficile* colonisation in humans is age dependent. While asymptomatic carriage is common in infants less than three years of age it is rare in adults [75]. As such, infants can be a major reservoir of both pathogenic and toxin-negative strains in a community setting [75]. We isolated five toxin-negative isolates of *C. difficile* including three from humans (22.1, 19.3, 5.3), one from a pig (P29) and one (H3) from a horse all showing symptoms of gastrointestinal disease. Despite efforts to identify *C. difficile* toxin genes or toxin gene products in the stool samples during the course of the isolation of these strains, none were detected. Phylogenetic studies showed that human ST39 isolates 19.3 and 22.1 and porcine ST109 isolate P29 grouped with clinical human toxigenic strains of ST39 and ST109 respectively in Clade 4. Furthermore, human ST15 isolate 5.3 and equine ST29 isolate H3 grouped with human clinical toxigenic strains with ST15 and ST29 respectively in Clade 1. Comparative genome analyses showed that our toxin-negative isolates displayed virulence gene profiles akin to those identified in toxigenic strains. The animals from which samples were collected in this study exhibited gastrointestinal disease and we were unable to attribute these symptoms to the presence of toxin-positive strains of *C. difficile*. Given the mobility of the PaLoc [44] and evidence that the acquisition or loss of the PaLoc via recombination [17] has occurred multiple times during the evolution of the five major clades of *C. difficile* [11, 17], our data reinforces calls to include toxin-negative strains in genomic epidemiological studies of *C. difficile* [17, 36] and to better characterise asymptomatic carriage of closely related *Clostridia* in gut microbiome surveys such as the Human Microbiome Project and MetaHIT, both in humans and close animal contacts.

Our reference-based whole genome alignment and phylogeny analyses support the global population structure of *C. difficile* as described by Dingle et al. in 2014 [14, 17, 19]. Each clade has been shown previously to have representatives of both toxin-positive and toxin-negative strains [11]. Our toxin-negative isolates (19.3, 22.1, 5.3, P29, H3) belonged to STs that are distinct from those reported in an earlier study [17]. The role of toxins in *C. difficile* infection has been extensively studied but factors that enable *C. difficile* to efficiently colonise the human gastrointestinal tract are relatively poorly understood and are not associated with genes encoded on the PaLoc. It is not known why some toxigenic strains evolve into dominant hypervirulent clones. Thus, considering the genetic diversity inherent within the phylogenetic structure of *C. difficile* [14] a sub-population of toxin-negative strains of *C. difficile* that are efficient colonisers of the host gastrointestinal tract may readily acquire the PaLoc and evolve to become future hypervirulent strains. Several proteins have been suggested to play crucial roles in the colonization of gastrointestinal epithelium and disease progression [43, 71, 73, 74, 76–78]. A recent global proteome study of *C. difficile* strains CD630 and R20291 has identified numerous extracellular proteins from culture supernatants that may contribute to the virulence attributes of these strains [70].

Our study reinforced the important role played by phage in the evolution of *C. difficile*. While PHAST analysis was useful for identifying phage sequences, the analysis may not have identified the full extent of lysogenic phage because our draft genomes remain in multiple scaffolds. Although the complete sequence of phage phiC2 was identified in isolates 19.3 and 22.1 the regions that had significant homology with phiC2 in isolates P29 and H3 were located on different scaffolds. We used a scaffold tiling approach against the closed genome of a reference strain to create the input file for PHAST analysis (PHAST converts the scaffolded genomes into a concatenated artificial chromosome prior to predicting the phage content) and as such it remains a possibility that the partial matches are a consequence of the data handling process. Phage phiC2 is present in the majority of human isolates of *C. difficile* [68]. However, we detected regions of phiC2 in strains P29 and H3 suggesting that further studies are needed to address issues surrounding the association of phiC2 in *C. difficile* of animal origin. We also identified the *C. difficile* temperate bacteriophage phiCD6356 from the Siphoviridae family in isolates P29 and H3 but not in our human isolates of *C. difficile*. Genomes of bacteriophages belonging to the Siphoviridae family range in size from 14 to 50 kb [79, 80] and this broad range may be a reflection of the stringency governing the amount of DNA that can be packaged by phiCD6356. In addition to the acquisition of phage-associated genes, a 119.3-kb region on contig 11 in isolate P29 was also identified in the course of this analysis. This region is unique to the P29 genome and displayed significant DNA sequence identity to portions of the CD630 genome. It remains unknown if the 119.3-kb region exists in *C. difficile* strains of porcine origin. Further analyses with greater numbers of genomes from both human and animal sources are required to conclusively address these questions.

## Conclusions

Our studies reinforce calls to improve our understanding of the physiological conditions that promote lateral transfer of the PaLoc in the gastrointestinal tract [44]. This is important because the conditions that facilitate movement of fragments of DNA carrying the PaLoc and their recombination into the chromosome are also conducive to the movement of conjugative transposons that carry antibiotic resistance genes and putative virulence factors as independent genetic events [44].

### Data accessibility

Genome sequences reported in this analysis were submitted to GenBank and are available via the accession numbers provided. The bioinformatics softwares are made available through the GitHub repository links.

## Additional files

Additional file 1: Table S1. Isolation history, genomic assembly and initial PCR screening results of the five isolates analysed in this manuscript. (DOCX 36 kb) Additional file 2: Figure S2. Preliminary phylogeny with reference genomes to identify the most closely related reference genome for tiling of genomic scaffolds. **Figure S3.** Mauve alignments of genomes 19.3 and 22.1. **Figure S7.** DNA sequence alignment of repetitive regions in PaLoc of isolates in clade 1. **Figure S10.** Phage profiles in the genomes of the five Australian toxin-negative C. difficile isolates included in this study. **Figure S12.** Preliminary Phylogenetic tree with all isolates included in Dingle et al’s publication in 2011 [11]. Isolates that form clade C-I is highlighted in blue. (DOCX 2963 kb) Additional file 3: Table S4. SNPs identified by Mauve alignments of genomes of C. difficile strains 19.3 and 22.1. **Table S5.** Bidirectional BLASTp analysis of peptide sequences predicted from genomes of C. difficile strains 19.3 and 22.1 to identify amino acid changes caused by SNPs in the genomes. **Table S8.** Assembly Statistics downloaded from short read archives. **Table S9.** Unique proteins identified from the All Vs All BLASTp analysis of C. difficile genomes. (XLSX 632 kb) Additional file 4: Table S11. Regions identified in each C. difficile genome by PHAST. Sheet 1. C. difficile strain P29 genome, Sheet 2. C. difficile strain H3 genome, Sheet 3. C. difficile strain 5.3 genome, Sheet 4. C. difficile strain 19.3 genome. Sheet 5. C. difficile strain 22.1 genome. (XLSX 134 kb) Additional file 5: Table S6. Sheet 1 = Comparative BLASTp analysis of 4 genomes, Sheet 2 = Alignment of scaffold 11.1 of genome P29 against C. difficile CD630 genome. (XLSX 682 kb)

## References

[1] Karadsheh Z, Sule S (2013). Fecal transplantation for the treatment of recurrent clostridium difficile infection. N Am J Med Sci.

[2] Lessa FC, Mu Y, Bamberg WM, Beldavs ZG, Dumyati GK, Dunn JR (2015). Burden of Clostridium difficile infection in the United States. N Engl J Med.

[3] Peterson LR (2009). Bad bugs, no drugs: no ESCAPE revisited. Clin Infect Dis.

[4] Redelings MD, Sorvillo F, Mascola L (2007). Increase in Clostridium difficile-related mortality rates, United States, 1999–2004. Emerg Infect Dis.

[5] Rupnik M, Wilcox MH, Gerding DN (2009). Clostridium difficile infection: new developments in epidemiology and pathogenesis. Nat Rev Microbiol.

[6] Khanna S, Pardi DS, Aronson SL, Kammer PP, Baddour LM (2012). Outcomes in community-acquired Clostridium difficile infection. Aliment Pharmacol Ther.

[7] Bauer MP, Kuijper EJ (2015). Potential sources of Clostridium difficile in human infection. Infect Dis Clin North Am.

[8] Songer JG, Anderson MA (2006). Clostridium difficile: an important pathogen of food animals. Anaerobe.

[9] Hensgens MP, Keessen EC, Squire MM, Riley TV, Koene MG, de Boer E (2012). Clostridium difficile infection in the community: a zoonotic disease?. Clin Microbiol Infect.

[10] Goorhuis A, Debast SB, van Leengoed LA, Harmanus C, Notermans DW, Bergwerff AA (2008). Clostridium difficile PCR ribotype 078: an emerging strain in humans and in pigs?. J Clin Microbiol.

[11] Dingle KE, Griffiths D, Didelot X, Evans J, Vaughan A, Kachrimanidou M (2011). Clinical Clostridium difficile: clonality and pathogenicity locus diversity. PLoS One.

[12] Walk ST, Micic D, Jain R, Lo ES, Trivedi I, Liu EW (2012). Clostridium difficile ribotype does not predict severe infection. Clin Infect Dis.

[13] Cairns MD, Stabler RA, Shetty N, Wren BW (2012). The continually evolving Clostridium difficile species. Future Microbiol.

[14] Stabler RA, Dawson LF, Valiente E, Cairns MD, Martin MJ, Donahue EH (2012). Macro and micro diversity of Clostridium difficile isolates from diverse sources and geographical locations. PLoS One.

[15] Behroozian AA, Chludzinski JP, Lo ES, Ewing SA, Waslawski S, Newton DW (2013). Detection of mixed populations of Clostridium difficile from symptomatic patients using capillary-based polymerase chain reaction ribotyping. Infect Control Hosp Epidemiol.

[16] Waslawski S, Lo ES, Ewing SA, Young VB, Aronoff DM, Sharp SE (2013). Clostridium difficile ribotype diversity at six health care institutions in the United States. J Clin Microbiol.

[17] Dingle KE, Elliott B, Robinson E, Griffiths D, Eyre DW, Stoesser N (2014). Evolutionary history of the Clostridium difficile pathogenicity locus. Genome Biol Evol.

[18] Monot M, Eckert C, Lemire A, Hamiot A, Dubois T, Tessier C (2015). Clostridium difficile: New insights into the evolution of the pathogenicity locus. Sci Rep.

[19] Griffiths D, Fawley W, Kachrimanidou M, Bowden R, Crook DW, Fung R (2010). Multilocus sequence typing of Clostridium difficile. J Clin Microbiol.

[20] Lemee L, Bourgeois I, Ruffin E, Collignon A, Lemeland JF, Pons JL (2005). Multilocus sequence analysis and comparative evolution of virulence-associated genes and housekeeping genes of Clostridium difficile. Microbiology.

[21] Lemee L, Dhalluin A, Pestel-Caron M, Lemeland JF, Pons JL (2004). Multilocus sequence typing analysis of human and animal Clostridium difficile isolates of various toxigenic types. J Clin Microbiol.

[22] Lemee L, Dhalluin A, Testelin S, Mattrat MA, Maillard K, Lemeland JF (2004). Multiplex PCR targeting tpi (triose phosphate isomerase), tcdA (Toxin A), and tcdB (Toxin B) genes for toxigenic culture of Clostridium difficile. J Clin Microbiol.

[23] Rupnik M (2001). How to detect Clostridium difficile variant strains in a routine laboratory. Clin Microbiol Infect.

[24] Voth DE, Ballard JD (2005). Clostridium difficile toxins: mechanism of action and role in disease. Clin Microbiol Rev.

[25] Hundsberger T, Braun V, Weidmann M, Leukel P, Sauerborn M, von Eichel-Streiber C (1997). Transcription analysis of the genes tcdA-E of the pathogenicity locus of Clostridium difficile. Eur J Biochem/FEBS.

[26] Matamouros S, England P, Dupuy B (2007). Clostridium difficile toxin expression is inhibited by the novel regulator TcdC. Mol Microbiol.

[27] Braun V, Hundsberger T, Leukel P, Sauerborn M, von Eichel-Streiber C (1996). Definition of the single integration site of the pathogenicity locus in Clostridium difficile. Gene.

[28] Eckert C, Emirian A, Le Monnier A, Cathala L, De Montclos H, Goret J (2015). Prevalence and pathogenicity of binary toxin-positive Clostridium difficile strains that do not produce toxins A and B. New Microbes New Infect.

[29] Collins DA, Elliott B, Riley TV (2015). Molecular methods for detecting and typing of Clostridium difficile. Pathology.

[30] Rupnik M, Brazier JS, Duerden BI, Grabnar M, Stubbs SL (2001). Comparison of toxinotyping and PCR ribotyping of Clostridium difficile strains and description of novel toxinotypes. Microbiology.

[31] Villano SA, Seiberling M, Tatarowicz W, Monnot-Chase E, Gerding DN (2012). Evaluation of an oral suspension of VP20621, spores of nontoxigenic Clostridium difficile strain M3, in healthy subjects. Antimicrob Agents Chemother.

[32] Nagaro KJ, Phillips ST, Cheknis AK, Sambol SP, Zukowski WE, Johnson S (2013). Nontoxigenic Clostridium difficile protects hamsters against challenge with historic and epidemic strains of toxigenic BI/NAP1/027 C. difficile. Antimicrob Agents Chemother.

[33] Natarajan M, Walk ST, Young VB, Aronoff DM (2013). A clinical and epidemiological review of non-toxigenic Clostridium difficile. Anaerobe.

[34] Seal D, Borriello SP, Barclay F, Welch A, Piper M, Bonnycastle M (1987). Treatment of relapsing Clostridium difficile diarrhoea by administration of a non-toxigenic strain. Eur J Clin Microbiol.

[35] Wilson KH, Sheagren JN (1983). Antagonism of toxigenic Clostridium difficile by nontoxigenic C. difficile. J Infect Dis.

[36] Buckley AM, Spencer J, Maclellan LM, Candlish D, Irvine JJ, Douce GR (2013). Susceptibility of hamsters to Clostridium difficile isolates of differing toxinotype. PLoS One.

[37] Sambol SP, Merrigan MM, Tang JK, Johnson S, Gerding DN (2002). Colonization for the prevention of Clostridium difficile disease in hamsters. J Infect Dis.

[38] Hung YP, Lin HJ, Wu TC, Liu HC, Lee JC, Lee CI (2013). Risk factors of fecal toxigenic or non-toxigenic Clostridium difficile colonization: impact of Toll-like receptor polymorphisms and prior antibiotic exposure. PLoS One.

[39] Gerding DN, Johnson S, Rupnik M, Aktories K (2014). Clostridium difficile binary toxin CDT: mechanism, epidemiology, and potential clinical importance. Gut Microbes.

[40] Geric B, Carman RJ, Rupnik M, Genheimer CW, Sambol SP, Lyerly DM (2006). Binary toxin-producing, large clostridial toxin-negative Clostridium difficile strains are enterotoxic but do not cause disease in hamsters. J Infect Dis.

[41] Bacci S, Molbak K, Kjeldsen MK, Olsen KE (2011). Binary toxin and death after Clostridium difficile infection. Emerg Infect Dis.

[42] Barbut F, Decre D, Lalande V, Burghoffer B, Noussair L, Gigandon A (2005). Clinical features of Clostridium difficile-associated diarrhoea due to binary toxin (actin-specific ADP-ribosyltransferase)-producing strains. J Med Microbiol.

[43] Barketi-Klai A, Monot M, Hoys S, Lambert-Bordes S, Kuehne SA, Minton N (2014). The flagellin FliC of Clostridium difficile is responsible for pleiotropic gene regulation during in vivo infection. PLoS One.

[44] Brouwer MS, Roberts AP, Hussain H, Williams RJ, Allan E, Mullany P (2013). Horizontal gene transfer converts non-toxigenic Clostridium difficile strains into toxin producers. Nat Commun.

[45] Casey TA, Bosworth BT (2009). Design and evaluation of a multiplex polymerase chain reaction assay for the simultaneous identification of genes for nine different virulence factors associated with Escherichia coli that cause diarrhea and edema disease in swine. J Vet Diagn Investig.

[46] Rinttila T, Kassinen A, Malinen E, Krogius L, Palva A (2004). Development of an extensive set of 16S rDNA-targeted primers for quantification of pathogenic and indigenous bacteria in faecal samples by real-time PCR. J Appl Microbiol.

[47] Darling AE, Worden P, Chapman TA, Roy Chowdhury P, Charles IG, Djordjevic SP (2014). The genome of Clostridium difficile 5.3. Gut pathogens.

[48] Darling AE, Jospin G, Lowe E, Matsen FA, Bik HM, Eisen JA (2014). PhyloSift: phylogenetic analysis of genomes and metagenomes. PeerJ.

[49] Li H, Handsaker B, Wysoker A, Fennell T, Ruan J, Homer N (2009). The Sequence Alignment/Map format and SAMtools. Bioinformatics.

[50] Schloss PD, Westcott SL, Ryabin T, Hall JR, Hartmann M, Hollister EB (2009). Introducing mothur: open-source, platform-independent, community-supported software for describing and comparing microbial communities. Appl Environ Microbiol.

[51] Bertels F, Silander OK, Pachkov M, Rainey PB, van Nimwegen E (2014). Automated reconstruction of whole-genome phylogenies from short-sequence reads. Mol Biol Evol.

[52] Stamatakis A (2006). RAxML-VI-HPC: maximum likelihood-based phylogenetic analyses with thousands of taxa and mixed models. Bioinformatics.

[53] Lartillot N, Philippe H (2004). A Bayesian mixture model for across-site heterogeneities in the amino-acid replacement process. Mol Biol Evol.

[54] Stamatakis A (2006). Phylogenetic models of rate heteroginity: a high performance computing perspective. Parallel and Distributed Processing Symposium, 2006.

[55] Stamatakis A, Hoover P, Rougemont J (2008). A rapid bootstrap algorithm for the RAxML Web servers. Syst Biol.

[56] Aziz RK, Bartels D, Best AA, DeJongh M, Disz T, Edwards RA (2008). The RAST Server: rapid annotations using subsystems technology. BMC Genomics.

[57] Overbeek R, Olson R, Pusch GD, Olsen GJ, Davis JJ, Disz T (2014). The SEED and the Rapid Annotation of microbial genomes using Subsystems Technology (RAST). Nucleic Acids Res.

[58] Salichos L, Rokas A (2011). Evaluating ortholog prediction algorithms in a yeast model clade. PLoS One.

[59] Frith MC, Hamada M, Horton P (2010). Parameters for accurate genome alignment. BMC Bioinformatics.

[60] Frith MC, Wan R, Horton P (2010). Incorporating sequence quality data into alignment improves DNA read mapping. Nucleic Acids Res.

[61] Kielbasa SM, Wan R, Sato K, Horton P, Frith MC (2011). Adaptive seeds tame genomic sequence comparison. Genome Res.

[62] Blodel VD, Guillaume JL, Lambiotte R, Lefebvre E (2008). Fast unfolding of communities in large networks. J Stat Mech.

[63] Darling AE, Treangen TJ, Messeguer X, Perna NT (2007). Analyzing patterns of microbial evolution using the mauve genome alignment system. Methods Mol Biol.

[64] Rissman AI, Mau B, Biehl BS, Darling AE, Glasner JD, Perna NT (2009). Reordering contigs of draft genomes using the Mauve aligner. Bioinformatics.

[65] Sullivan MJ, Petty NK, Beatson SA (2011). Easyfig: a genome comparison visualizer. Bioinformatics.

[66] Zhou Y, Liang Y, Lynch KH, Dennis JJ, Wishart DS (2011). PHAST: a fast phage search tool. Nucleic Acids Res.

[67] Goh S, Chang BJ, Riley TV (2005). Effect of phage infection on toxin production by Clostridium difficile. J Med Microbiol.

[68] Goh S, Ong PF, Song KP, Riley TV, Chang BJ (2007). The complete genome sequence of Clostridium difficile phage phiC2 and comparisons to phiCD119 and inducible prophages of CD630. Microbiology.

[69] Horgan M, O'Sullivan O, Coffey A, Fitzgerald GF, van Sinderen D, McAuliffe O (2010). Genome analysis of the Clostridium difficile phage PhiCD6356, a temperate phage of the Siphoviridae family. Gene.

[70] Cafardi V, Biagini M, Martinelli M, Leuzzi R, Rubino JT, Cantini F (2013). Identification of a novel zinc metalloprotease through a global analysis of Clostridium difficile extracellular proteins. PLoS One.

[71] Pettit LJ, Browne HP, Yu L, Smits WK, Fagan RP, Barquist L (2014). Functional genomics reveals that Clostridium difficile Spo0A coordinates sporulation, virulence and metabolism. BMC Genomics.

[72] Hennequin C, Porcheray F, Waligora-Dupriet A, Collignon A, Barc M, Bourlioux P (2001). GroEL (Hsp60) of Clostridium difficile is involved in cell adherence. Microbiology.

[73] Merrigan MM, Venugopal A, Roxas JL, Anwar F, Mallozzi MJ, Roxas BA (2013). Surface-layer protein A (SlpA) is a major contributor to host-cell adherence of Clostridium difficile. PLoS One.

[74] Baban ST, Kuehne SA, Barketi-Klai A, Cartman ST, Kelly ML, Hardie KR (2013). The role of flagella in Clostridium difficile pathogenesis: comparison between a non-epidemic and an epidemic strain. PLoS One.

[75] Rousseau C, Poilane I, De Pontual L, Maherault AC, Le Monnier A, Collignon A (2012). Clostridium difficile carriage in healthy infants in the community: a potential reservoir for pathogenic strains. Clin Infect Dis.

[76] Dawson LF, Valiente E, Faulds-Pain A, Donahue EH, Wren BW (2012). Characterisation of Clostridium difficile biofilm formation, a role for Spo0A. PLoS One.

[77] Deakin LJ, Clare S, Fagan RP, Dawson LF, Pickard DJ, West MR (2012). The Clostridium difficile spo0A gene is a persistence and transmission factor. Infect Immun.

[78] Ethapa T, Leuzzi R, Ng YK, Baban ST, Adamo R, Kuehne SA (2013). Multiple factors modulate biofilm formation by the anaerobic pathogen Clostridium difficile. J Bacteriol.

[79] Petrovski S, Dyson ZA, Seviour RJ, Tillett D (2012). Small but sufficient: the Rhodococcus phage RRH1 has the smallest known Siphoviridae genome at 14.2 kilobases. J Virol.

[80] Sekulovic O, Garneau JR, Neron A, Fortier LC (2014). Characterization of temperate phages infecting Clostridium difficile isolates of human and animal origins. Appl Environ Microbiol.

