# Supplementary material for: Comparative genomic analysis of toxin-negative strains of Clostridium difficile from humans and animals with symptoms of gastrointestinal disease
Source: BMC Microbiol. 2016 Mar 12;16:41. doi: 10.1186/s12866-016-0653-3 (PMC4789261; doi:10.1186/s12866-016-0653-3)
Supplement: Additional file 1: Table S1. — Isolation history, genomic assembly and initial PCR screening results of the five isolates analysed in this manuscript. (DOCX 36 kb) [file 12866_2016_653_MOESM1_ESM.docx]

**Additional file 1: Table S1**

| **Strain ID** | **P29** | **5.3** | **19.3** | **22.1** | **H3** |
| --- | --- | --- | --- | --- | --- |
| Source | Porcine | Human | Human | Human | Equine |
| Health Status | Scouring -alive/3day old | Diarrhoea | Diarrhoea | Diarrhoea | Scouring- alive/3day old |
| Year of Isolation | 2008 | 2008 | 2008 | 2008 | 2008 |
| **PCR Genotyping:** |  |  |  |  |  |
| Ribotyping primers | A | B | C | C | D |
| *tpi* gene amplicon | 230bp | 230bp | 230bp | 230bp | 230bp |
| *tcdA* | negative | negative | negative | negative | negative |
| *tcd B* | negative | negative | negative | negative | negative |
| **Sequence Quality analysis:** | |  |  |  |  |
| Total number of Reads | 1459536 | 1850340 | 1672156 | 1618370 | 2467620 |
| Average read lengths(nt) | 32-251 | 35-301 | 35-301 | 35-310 | 32-251 |
| Phred score | 38/35 | 29/24 | 30/23 | 30/23 | 39/35 |
| Per base N-content | 0 | 0 | 0 | 0 | 0 |
| Over-represented seq | None | None | None | None | None |
| **Assembly Statistics:** |  |  |  |  |  |
| Scaffold numbers | 74 | 27 | 65 | 58 | 76 |
| Median Coverage (folds) | 65 | 89 | 73 | 71 | 120 |
| Largest Scaffold size (nt) | 404355 | 1447561 | 438180 | 471213 | 485192 |
| Average Scaffold size (nt) | 58345.6 | 148493 | 64335.5 | 72084.4 | 54449.9 |
| Scaffold N50 (nt) | 215323 | 786725 | 178616 | 196766 | 217879 |
| Scaffold N90 (nt) | 57174 | 135837 | 61678 | 61855 | 87614 |
| GC content (%) | 29.09 | 28.32 | 28.77 | 28.76 | 28.75 |
| Size of Genome(nt) | 4317574 | 4009318 | 4181809 | 4180898 | 4138192 |
| **Bioinformatic analysis** |  |  |  |  |  |
| ST type | 109 | 15 | 39 | 39 | 29 |
| MLST clade | None defined | 1 | None defined | None defined | None defined |
| PaLoc | None | None | None | None | None |
| *cdtA* | None | None | None | None | None |
| *cdtB* | None | None | None | None | None |
