# Supplementary material for: Comparative genomic analysis of toxin-negative strains of Clostridium difficile from humans and animals with symptoms of gastrointestinal disease
Source: BMC Microbiol. 2016 Mar 12;16:41. doi: 10.1186/s12866-016-0653-3 (PMC4789261; doi:10.1186/s12866-016-0653-3)
Supplement: Additional file 2: Figure S2. — Preliminary phylogeny with reference genomes to identify the most closely related reference genome for tiling of genomic scaffolds. Figure S3. Mauve alignments of genomes 19.3 and 22.1. Figure S7. DNA sequence alignment of repetitive regions in PaLoc of isolates in clade 1. Figure S10. Phage profiles in the genomes of the five Australian toxin-negative C. difficile isolates included in this study. Figure S12. Preliminary Phylogenetic tree with all isolates included in Dingle et al’s publication in 2011 [11]. Isolates that form clade C-I is highlighted in blue. (DOCX 2963 kb) [file 12866_2016_653_MOESM2_ESM.docx]

**Additional file 2**

**Figure S2:** Preliminary phylogeny with reference genomes to identify the most closely related reference genome for tiling of genomic scaffolds.


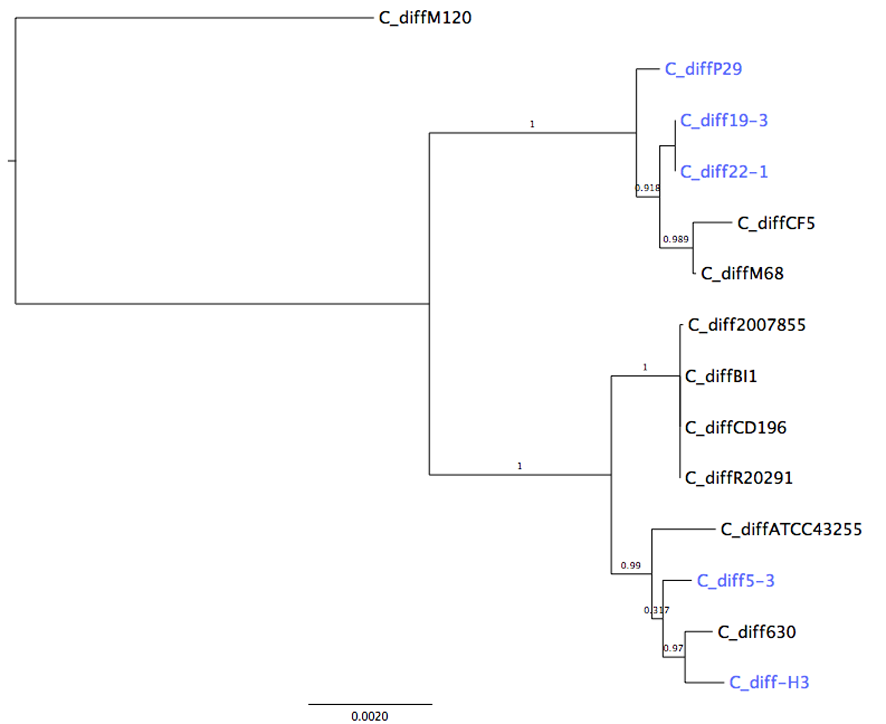


**Figure S3:** Mauve alignments of genomes 19.3 and 22.1.


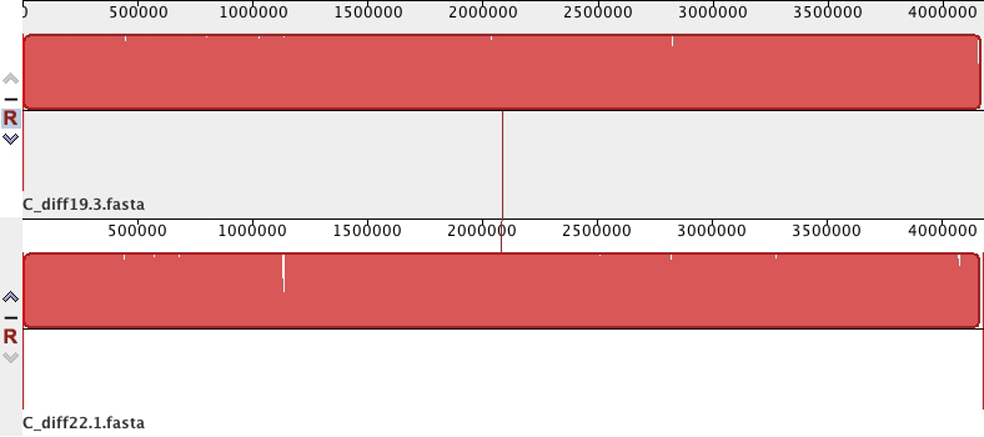


**Key:** Red indicates regions of sequence identity. White streaks indicate regions where nucleotide similarity drops.

**Figure S7:** DNA sequence alignment of repetitive regions in PaLoc of isolates in clade 1.

>HG002393

GAATATATTTTACAA**CATTATAGATTGTTTTAAATCTCTTTGAATATTCTACAAGACTATAGATTAGTTTAAATCCCTTT**GAATATTTCAAAACACCATAGATTACTTTAAAT

>Scaffold3.1_5.3

GAATATATTTTA**CAGCATTATAGATTGCTTTAAATCTCTTTGAGTATTCTACAAGACTATAGATTAGTTTAAATCCCTTTGAATATATTTTA**CAA**CATTATAGATTGTTTTAAATCTCTTTGAATATTCTACAAGACTATAGATTAGTTTAAATCCCTTT**GAATATTTCAAAACACCATAGATTACTTTAAAT

>Scaffold4.1_H3

GAATATATTTTACAG**CATTATAGATTG**C**TTTAAATCTCTTTGAATATTCTACAAGACTATAGATTAGTTTAAATCCCTTT**GAATATATTTTACAA**CATTATAGATTG**T**TTTAAATCTCTTTGAATATTCTACAAGACTATAGATTAGTTTAAATCCCTTT**GAATATATTTTACAA**CATTATAGATTGTTTTAAATCTCTTTGAATATTCTACAAGACTATAGATTAGTTTAAATCCCTTTCATTATAGATTG**CTTCAAAACACCATAGATTACTTTAAAT

Key:

Red and Blue regions = regions of sequence identity across the region in the 2 genomes in context to the reference in GenBank (ID HG002393).

Underlined regions = identical sequences within the insertions

Green highlight = 80nt insertion

Yellow highlight =68nt repeat sequence

Differently coloured bases within the highlighted regions indicate nucleotide differences.

**Figure S10:** Phage profiles in the genomes of the five Australian toxin-negative *C. difficile* isolates included in this study.


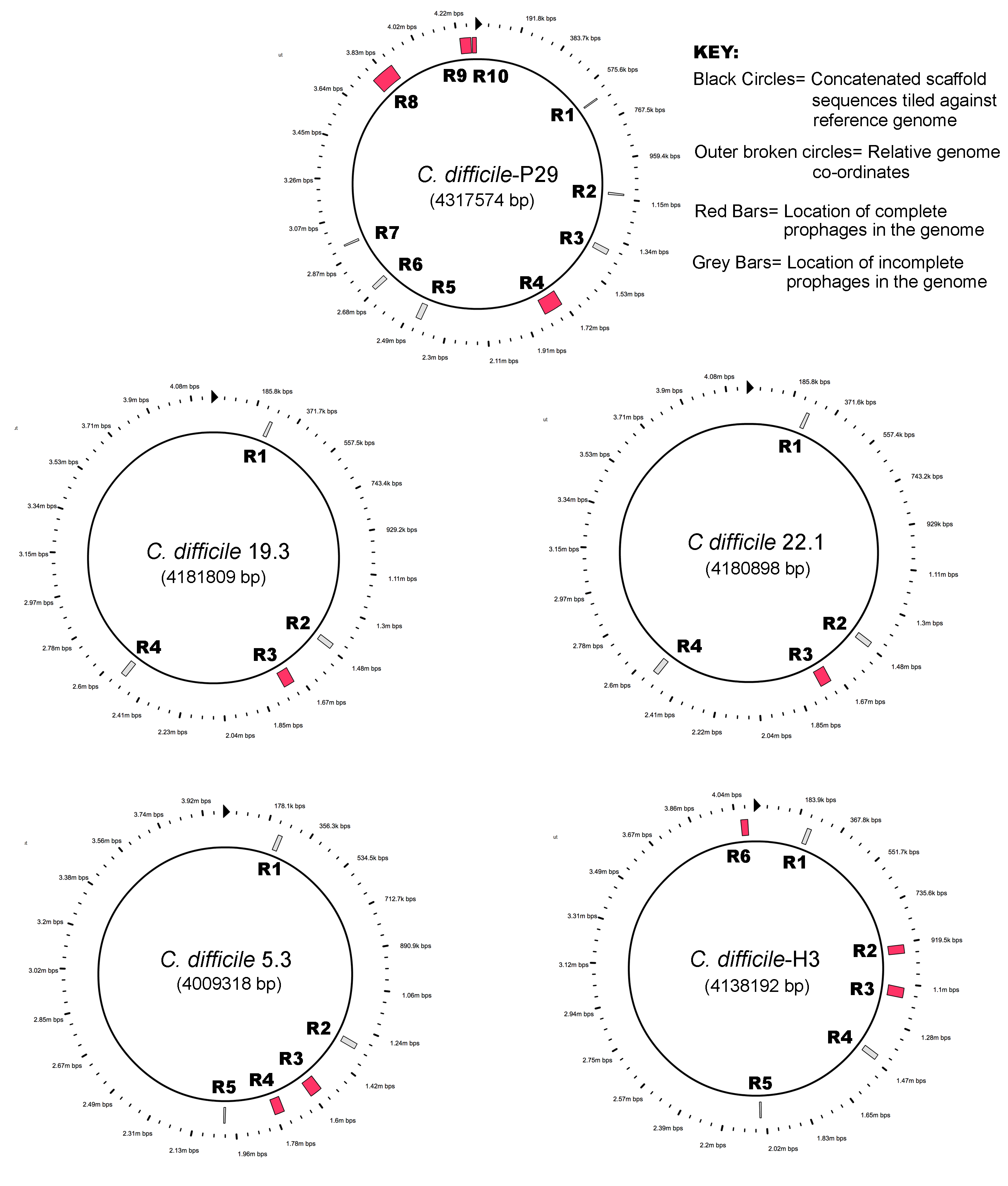


**Figure S12**: Preliminary Phylogenetic tree with all isolates included in Dingle et al’s publication in 2011 (ref 11). Isolates that form clade C-I is highlighted in blue.
